# Trans-generational inheritance of herbivory-induced phenotypic changes in *Brassica rapa*

**DOI:** 10.1038/s41598-018-21880-2

**Published:** 2018-02-23

**Authors:** Roman T. Kellenberger, Gaylord A. Desurmont, Philipp M. Schlüter, Florian P. Schiestl

**Affiliations:** 1 Department of Systematic and Evolutionary Botany, University of Zurich, Zollikerstrasse 107, CH-8008 Zurich, Switzerland; 2 Institute of Biology, University of Neuchâtel, Avenue du 1er-Mars 26, CH-2000 Neuchâtel, Switzerland; 3 EBCL USDA ARS, Campus international de Baillarguet, 34980 Montferrier sur lez, France

## Abstract

Biotic stress can induce plastic changes in fitness-relevant plant traits. Recently, it has been shown that such changes can be transmitted to subsequent generations. However, the occurrence and extent of transmission across different types of traits is still unexplored. Here, we assessed the emergence and transmission of herbivory-induced changes in *Brassica rapa* and their impact on interactions with insects. We analysed changes in morphology and reproductive traits as well as in flower and leaf volatile emission during two generations with leaf herbivory by *Mamestra brassicae* and *Pieris brassicae* and two subsequent generations without herbivory. Herbivory induced changes in all trait types, increasing attractiveness of the plants to the parasitoid wasp *Cotesia glomerata* and decreasing visitation by the pollinator *Bombus terrestris*, a potential trade-off. While changes in floral and leaf volatiles disappeared in the first generation after herbivory, some changes in morphology and reproductive traits were still measurable two generations after herbivory. However, neither parasitoids nor pollinators further discriminated between groups with different past treatments. Our results suggest that transmission of herbivore-induced changes occurs preferentially in resource-limited traits connected to plant growth and reproduction. The lack of alterations in plant-insect interactions was likely due to the transient nature of volatile changes.

## Introduction

To maximize their fitness, sessile organisms such as plants need to quickly respond to environmental stresses by readjusting the appropriate phenotypic traits^1–4^. In most cases, these environmentally induced changes are either transient and disappear in the next unstressed generation or short-term and reset at the latest two generations after induction^5,6^. Nevertheless, there are a few studies where the transmission of individual trait changes has been tracked long-term over more than one generation after stress treatment^7–10^. Despite these findings, the occurrence and relative importance of transient, short-term and long-term effects has not yet been systematically tested across multiple traits.

Communication with insect visitors is one of the most specific plant-environment interactions. Processes such as the attraction of pollinators or the deterrence of herbivores are mediated by orchestrated signalling of different trait types, e.g. visual perception of morphological traits and olfactory recognition of leaf and flower volatiles^11,12^. Changes such as the appearance of a new herbivore can thus not only lead to simultaneous shifts in many somatic and reproductive trait types^13–15^, but also create fundamentally different patterns of trait changes depending on the type of herbivore (e.g. a generalist vs. specialist species)^16^. However, measuring such changes is not trivial. In natural populations, genetic, epigenetic and environmental variation can mask phenotypic effects of induced changes^17,18^. On the other hand, studies conducted with (epi-)genetically uniform plants under controlled conditions often focus on particular traits and fail to identify the overall ecological significance of the induced changes^19^.

One of the best studied plant-insect relationships are the natural interactions of the crop plant *Brassica rapa* L. with both mutualistic and antagonistic insect species: Typically, *B. rapa* deters herbivores via the production of constitutive and inducible glucosinolate defence compounds^20^, but several herbivores such as the specialist *Pieris brassicae* L. or the generalist *Mamestra brassicae* L. are able to detoxify or tolerate these compounds^21,22^. To reduce damage caused by these herbivores, *B. rapa* relies on the attraction of specialized natural enemies such as parasitoid wasps (e.g. *Cotesia glomerata* L.) via herbivore-induced volatile organic compounds (VOCs)^23–25^. Simultaneously, herbivory has indirect effects on plant morphology and floral VOC production, altering the attractiveness of the plant to pollinators (e.g. *Bombus terrstris* L.)^26,27^. *B. rapa* is thus an ideal system to study the effect of herbivory-induced trait changes on interactions with other insect visitors.

Here, we assessed transient, short-term and long-term changes in *B. rapa* subjected to leaf herbivory by the specialist *P. brassicae* and generalist *M. brassicae*. Employing a multi-generational approach with minimized standing (epi-)genetic variation and without selection, the objectives of our study were to: 1. measure the induction of trait changes in plant morphology, seed production and the emission of leaf and flower volatiles under one and two generations of herbivory, 2. determine whether and which of the induced changes are transient or retained one and two generations after herbivory and 3. assess the effects of these changes on indirect defence and pollinator attraction in bioassays with parasitoids (*C. glomerata*) and pollinators (*B. terrestris*).

## Results

### Induction and retention of phenotypic changes upon herbivory

**Figure 1:**
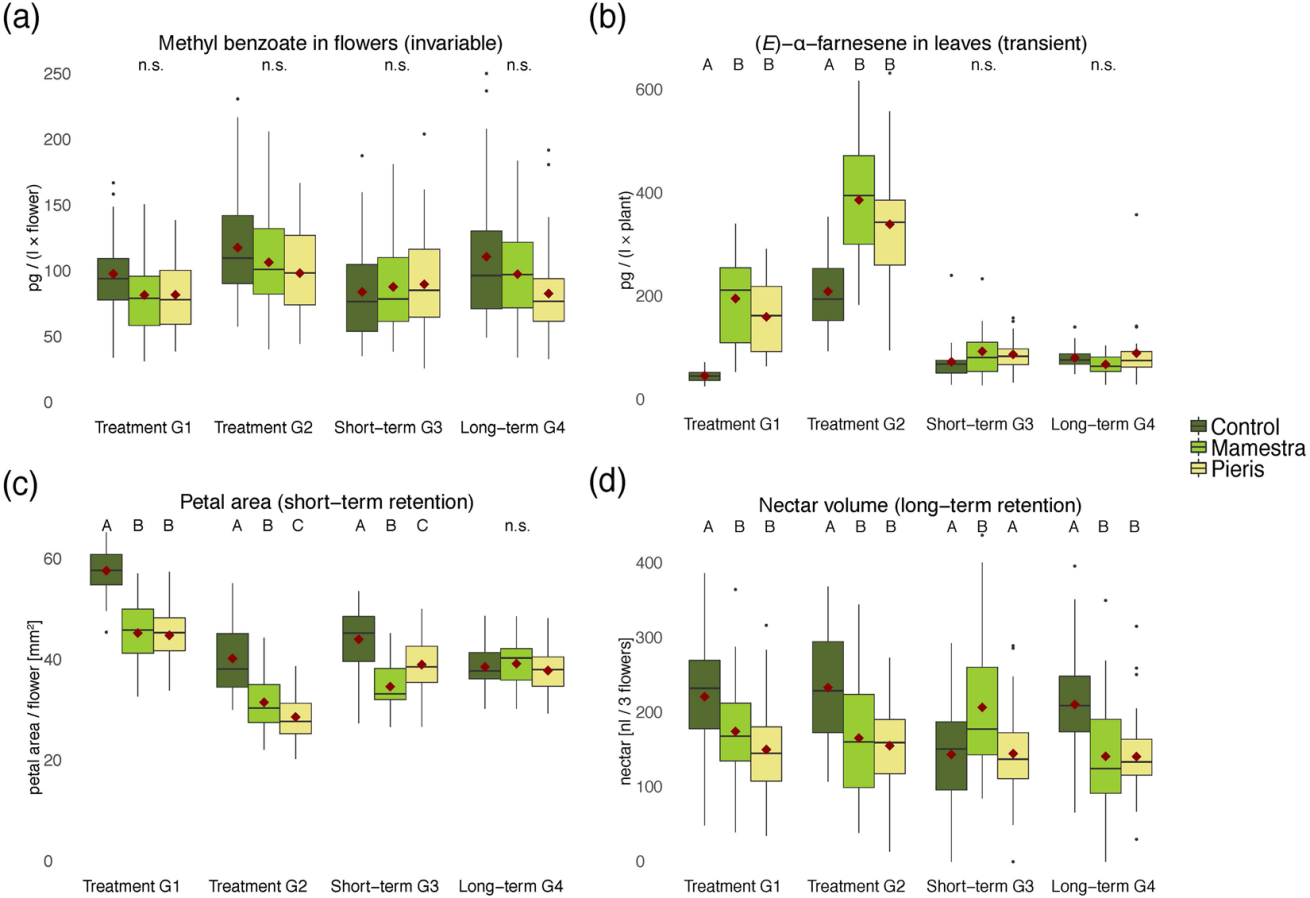
Examples of traits from all four response types. Letters above boxes denote group differences within generation (two-way ANOVA with *post hoc* Tukey HSD), red diamonds denote group means. (**a**) Invariable response; emission of methyl benzoate. (**b**) Transient response; emission of (*E*)-α-farnesene. (**c**) Short-term response; reduction in petal size in both treatment groups. (**d**) Long-term response; reduction in nectar volume.

Within the four plant generations, four different responses to herbivory were recorded across all twenty-two measured traits (Fig. 1a–d): While seven traits were invariable, herbivory by *Pieris brassicae* and *Mamestra brassicae* induced transient changes in six, short-term effects in three and long-term effects in five traits. One trait (the VOC methyl salicylate) showed an irregular pattern with only the first and last generation affected (see Table 1, Supplementary Table S1 and Supplementary Fig. S1).

**Table 1:** Summarised MANOVA and two-way ANOVA analyses of trait changes upon herbivory.

	Treatment g1		Treatment g2		Short-term g3		Long-term g4	
	*F-*value	DF	*F-*value	DF	*F-*value	DF	*F-*value	DF
**Morphological traits**	7.79***	6, 112	10.85***	6, 110	7.00***	7, 105	3.34***	5, 110
Plant height [cm]	5.19	—	4.45	—	1.61	—	0.36	—
Flower number	2.51	—	3.50	—	3.07	—	1.63	—
Bud number	0.68	—	9.06**	**M↑**	12.65***	**M↑**	9.43**	**MP↓**
Leaf number	0.64	—	0.74	—	18.72***	**M↑**	7.48*	**MP↓**
Nectar [nl/3 flowers]	9.82**	**MP↓**	13.50***	**MP↓**	8.63**	**M↑**	13.61***	**MP↓**
Petal area per flower [mm^2^]	83.59***		47.01***	**MP↓**	39.23***	**MP↓**	1.27	—
Total petal area [mm^2^]	27.84***	**MP↓**	21.03***	**MP↓**	9.25**	**MP↓**	2.15	—
**Reproductive traits**	1.36	6, 112	6.14***	6, 111	3.51***	7, 105	4.66***	5, 110
Silique number	n.a.	—	1.29	—	8.06**	**MP↑**	11.79***	**MP↑**
Seed weight [mg/10 seeds]	n.a.	—	5.29	—	6.56*	**M↑**	6.20*	**MP↓**
Seed number	n.a.	—	2.92	—	6.26*	**M↑**	5.76	—
Seed viability [%]	n.a.	-	6.37*	**M↓**	2.65	—	3.17	—
**Leaf VOC [pg/l]**	8.01***	2, 33	12.76***	2, 74	0.68	2, 54	2.48*	2, 73
1-Butene-4-isothiocyanate	2.38	—	14.48***	**MP↑**	n.a.	—	4.90	—
Benzyl nitrile	10.99**	**MP↑**	11.57***	**MP↑**	n.a.	—	1.74	—
(*E*)-α-Farnesene	28.75***	**MP↑**	20.30***	**MP↑**	n.a.	—	2.71	—
Flower VOC [pg/flower*l]	2.95***	6, 112	2.54**	6, 111	2.65***	7, 105	3.90***	5, 110
Benzaldehyde	4.33	—	3.62	—	0.37	—	2.93	—
1,3,5-Trimethylbenzene	4.81	—	3.03	—	1.06	—	3.00	—
Phenylacetaldehyde	3.28	—	0.75	—	1.82	—	1.05	—
Methyl benzoate	4.39	—	3.74	—	0.42	—	5.15	—
Benzyl nitrile	5.42	—	9.19**	**P↓**	1.33	—	3.46	—
Methyl salicylate	13.06***	**MP↓**	2.98	—	2.71	—	16.07***	**MP↓**
(*Z*)-α-Farnesene	3.89	—	0.10	—	0.05	—	3.27	—
(*E*)-α-Farnesene	8.73**	**MP↓**	6.75*	**P↓**	0.86	—	3.97	—

Significance levels: *P < 0.05; **P < 0.01; ***P < 0.001. “DF”: degrees of freedom within and between groups per generation and trait type. “M”: effect on *M. brassicae* group, “P”: effect on *P. brassicae* group. Arrow up (↑): increase in trait value, arrow down (↓): decrease in trait value, minus (−): no change in trait value, “n.a.”: no ANOVA calculated due to non-significant MANOVA result (*P ≥ *0.05).

Responses varied strongly between trait types: While transient effects were mostly present among leaf and flower VOCs, short and long-term effects were exclusively recorded among morphological and reproductive traits and completely absent among VOCs. Two generations of herbivory transiently increased mean emission of all three leaf VOCs between 35% and 202% (*M. brassicae*) and between 11% and 66% (*P. brassicae*). Simultaneously, mean emission of four flower VOCs was transiently decreased between 25% and 26% and between 21% and 38%. One generation after treatment, plants still had a 21% (*M. brassicae*) and 11% (*P. brassicae*) smaller petal area per flower, but *M. brassicae*-treated plants produced 25% more seeds. Two generations after treatment, plants had significantly fewer buds (*M. brassicae*: 13% and *P. brassicae*: 15%), fewer leaves (9% and 9%) and less nectar (33% and 33%). In addition, plants had significantly smaller seeds (7% and 6%), but an increased number of siliques (17% and 27%, see Table 1, Supplementary Table S1 and Supplementary Fig. S1). In plants from both treatment groups, significantly more flowers (open flowers + bud number) developed into siliques two generations after herbivory (two-way ANOVA, *F* (2,114) = 32.21, *P* < 0.001 and *post hoc* Tukey HSD, *P* (*P. brassicae* - control) <0.001, *P* (*M. brassicae* - control) <0.001, *P* (*P. brassicae* - *M. brassicae*) = 0.142).

In most cases, induction and direction of trait changes caused by the generalist herbivore *M. brassicae* did not differ from those caused by the specialist *P. brassicae*. However, in *M. brassicae-*treated plants, some morphological and reproductive traits were additionally affected in generation 2 (number of buds and seed viability) and 3 (number of buds, leaves, nectar and seeds, as well as seed weight). On the other hand, *P. brassicae-*treated plants showed an additional transient reduction of the two flower VOCs benzyl nitrile and (*E*)-α-farnesene. Although the background level of the control group was similar across generations for most traits, there were still some noticeable differences: Leaf VOC emission increased in the second treatment generation and petal size decreased within all four plant generations.

**Figure 2:**
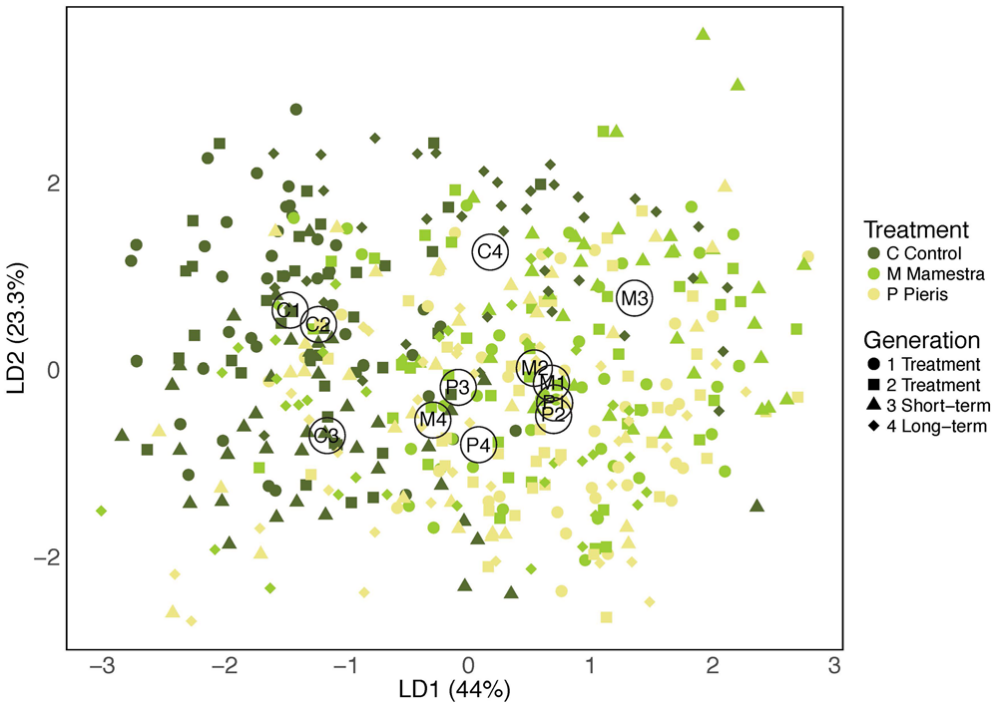
Result of the linear discriminant analysis (Wilks’ Λ (453,11): 0.242, *F* = 3.387, *P* < 2.2 × 10^−16^). The first two dimensions (LD1 and LD2) explain 44.0% and 23.3% of the observed group variance (see Supplementary Table S2 for loadings of LD1 and LD2). The analysis was conducted with ranked and z-transformed data of all measured traits except total petal area and leaf VOCs. Letters indicate centroid positions of *Mamestra brassica* (M) and *Pieris brassicae* (P) treated plant groups as well as the control groups (C) of generation 1–4.

Linear discriminant analysis showed a significant separation of groups across generations and treatments (Wilks’ Λ (453,11): 0.242, *F* = 3.387, *P* < 2.2 × 10^−16^). Treatment and control groups were largely separated in the first two linear discriminant dimensions, which explained 44.0% and 23.3% of the observed group variance. However, while the phenotypes of *M. brassicae* and *P. brassicae*-treated plants were indistinct from each other during treatment generations 1 and 2, both variance and separation increased in all treatment groups after treatment in generations 3 and 4. (Fig. 2, Supplementary Table S2).

### Attractiveness of herbivore-damaged plants to parasitoids and pollinators

**Figure 3:**
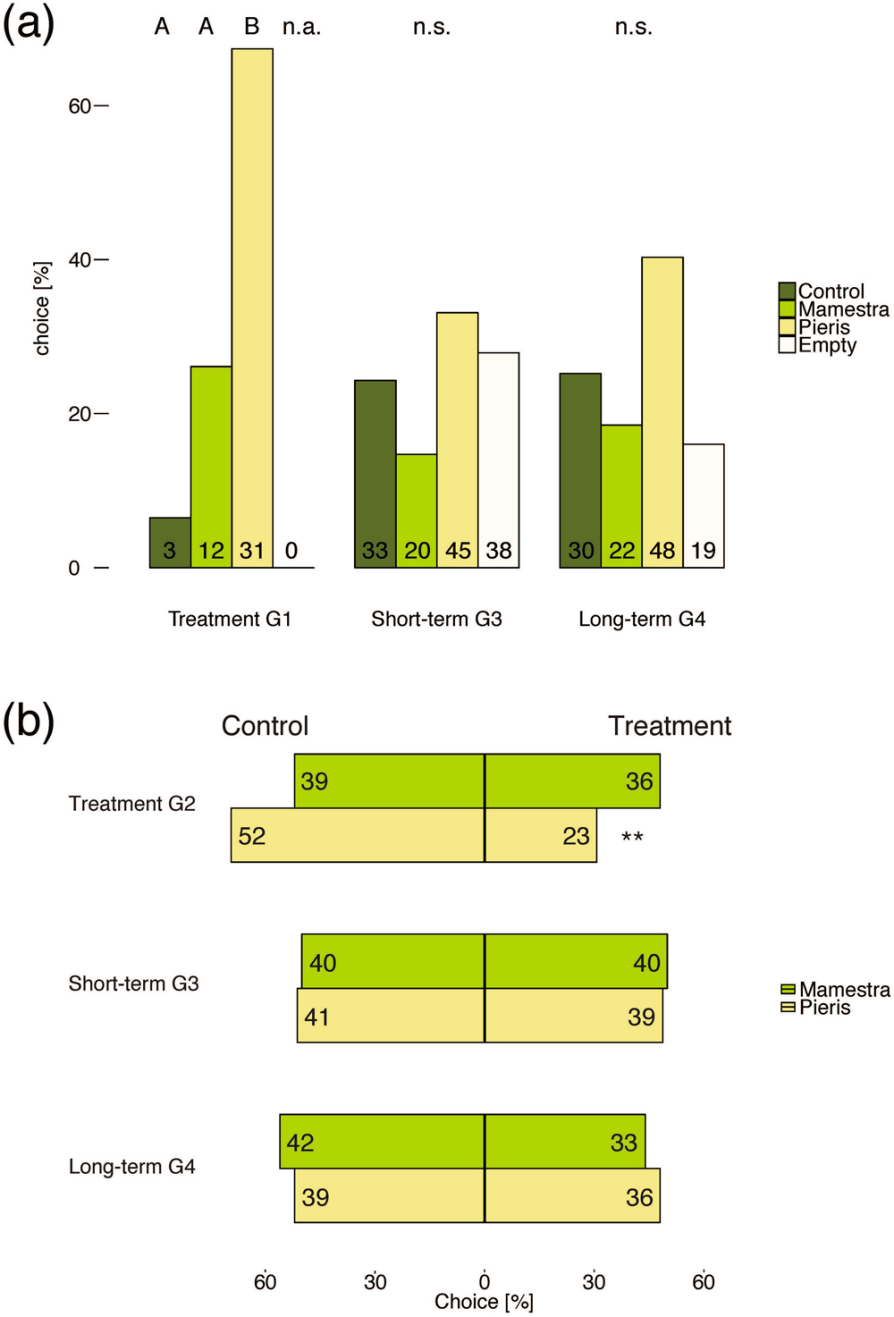
Results of the bioassays with parasitoids and pollinators. **(a)** Olfactometer tests with the parasitoid wasp *C. glomerata*. Numbers in bars indicate the absolute amount of wasps in this arm and letters above boxes denote group differences within generation (negative binomial mixed model with *post hoc* Tukey HSD excluding the empty arm due to complete separation, *Z* (Control-Pieris) = 3.806, *P* (Control-Pieris) <0.001). **(b)** Results of the dual-choice assays with the pollinator *B. terrestris* (binomial test, *P* = 0.001).

The choices made by *Cotesia glomerata* parasitoids were examined with a four-arm olfactometer containing one plant from each treatment and one empty arm. Among plants from generation 1, parasitoids had a significant preference for plants damaged by *P. brassicae*, which is their natural host. Also, none of the wasps chose the empty arm (Fig. 3a). In contrast, no significant choice could be detected among plants from the first and second generation (generations 3 and 4) without treatment, using statistical models either including or excluding the empty arm (Fig. 3a, also see methods). In generations 3 and 4, fewer wasps remained undecided in the central chamber, but some wasps entered the empty arm, which is also an indicator for a lack of a clear signal from one of the arms. In the dual-choice assays, plants subjected to *P. brassicae* herbivory were significantly less attractive to the pollinator *Bombus terrestris* in the treatment generation, but this effect disappeared in the two generations after the treatment. Pollinators did not discriminate between plants subjected to *M. brassicae* herbivory and control plants in all three generations (Fig. 3b).

## Discussion

Using a highly inbred *Brassica rapa* line, we investigated the induction and multigenerational transmission of herbivory-induced phenotypic changes, as well as their impact on plant-insect interactions. Under direct herbivory, changes among plant morphology and volatiles were clearly detectable, reduced pollinator attraction and induced attraction of parasitoids. After cessation of herbivory, some changes in plant morphology and reproductive traits were retained in one or even two following plant generations, supporting the presence of a trans-generational transmission of some environmentally induced changes. While the potential adaptive nature of these changes is not yet clear, the transient nature of the observed shifts in parasitoid- and pollinator attraction, likely mediated through volatiles, is expected to be advantageous: A retention of these signals would be maladaptive as they would give dishonest cues to parasitoids^28^ and maintain a trade-off unfavourable for pollinator attraction. We suggest that the action of natural selection on the transmission of induced changes results in a mosaic-like pattern of transient and retained phenotypic alterations.

Previous work has not only demonstrated that herbivory can alter pollinator-relevant traits of *B. rapa* and other plants, but also identified trade-offs between indirect defence and pollinator attraction^26,29–31^. Altogether, the phenotypic shifts observed in this study are consistent with such a trade-off: Nectar production and petal size, which were both decreased under herbivory treatment, are not only costly in their production^32–34^ but also have a great impact on pollinator attraction^35,36^. Also, petal size was previously found to be reduced in *B. rapa* under herbivore attack^27^. Simultaneously, herbivory increased the emission of all three measured leaf VOCs: 1-butene-4-isothiocyanate is a glucosinolate derivative well-known for its anti-herbivore properties^37,38^ and both benzyl nitrile and (*E*)-α-farnesene are reported to be inducible upon herbivory in *B. rapa*^23^. Studies have shown that 1-butene-4-isothiocyanate as well as benzyl nitrile are attractive to *Cotesia* parasitoids^23,39^, which may at least partly explain their preference for *P. brassicae*-damaged plants in the first generation of this study. In contrast, floral VOC emission was much less affected, which is congruent with findings of a previous study using another *B. rapa* inbred line (Kellenberger, *et al*.^40^, but see Schiestl, *et al*.^26^ using wild type *B. rapa* plants). However, the compound (*E*)-α-farnesene has been shown to be positively correlated with reward and elicits responses in *B. terrestris* olfactory neurons^41^. The observed reduction of this compound together with the smaller petal size may thus explain the reduced attractiveness of *P. brassicae-*infested plants to pollinators. Nevertheless, the general response of the *B. rapa* plants was similar for the specialist *P. brassicae* and the generalist *M. brassicae*, although it has been shown that caterpillar herbivores can elicit species-specific responses in host plants^42,43^.

So far, persistence of herbivore-induced phenotypic changes across generations has been documented mainly in the context of direct defence: It has been shown that herbivory can induce trans-generational changes in defensive plant traits such as trichomes^44^, cyanides^45^, glucosinolates^8^ and other phytochemicals^46^. However, systematic screens also including non-defensive traits and plant volatiles have hardly been conducted. Trans-generational effects observed in this study were restricted to morphological and seed traits, with most of them showing a reduced performance. As visible in different trait levels of the control group across generations, unknown physiological or environmental factors also led to inter-generational variation in the overall performance of the plants, which might have co-influenced the magnitude of trait changes. However, many of the affected traits can be considered rather costly and experiments with *Pieris* have shown a similar reduction in biomass^8^ and seed size^47^ among descendants of herbivory-damaged Brassicaceae plants. The observed reduction could thus be either a negative effect resulting from the inferior performance of the treated ancestral plants or an adaptive reallocation of resources^48^. While our study did not look at direct defence mechanisms, it is plausible that resources might also be reallocated to glucosinolate biosynthesis in the progeny. In the last years, molecular mechanisms have been described which could provide a framework for the inheritance of such induced changes, including maternal effects^49–51^ and epigenetic mechanisms such as DNA methylation, histone modifications or RNAi^19,52,53^.

In the generations after treatment, changes in plant volatiles, which are important signals for insect attraction, were not transmitted to offspring after treatment. Also, both parasitoids and pollinators did not discriminate among the progeny of the herbivore-treated plant groups. Since a constitutive attraction of parasitoids would be a dishonest “cry wolf “ signal^28^, it can be assumed that the plastic nature of signalling effective to parasitoids is adaptive. This may also be the case for the reduced attraction of pollinators, which should be reset as soon as trade-offs between defence and attraction are released. Although our study did not show whether any of the retained phenotypic changes have an adaptive value, it provides some hints on possible fitness-relevant properties: Unlike in control plants where later-developing flowers usually do not produce siliques anymore, seed production in progeny of herbivore-treated plants is distributed over the whole flowering time and among the majority of flowers. This interesting shift in resource allocation should be further evaluated in future studies.

## Material and Methods

### Plants and treatments

**Figure 4:**
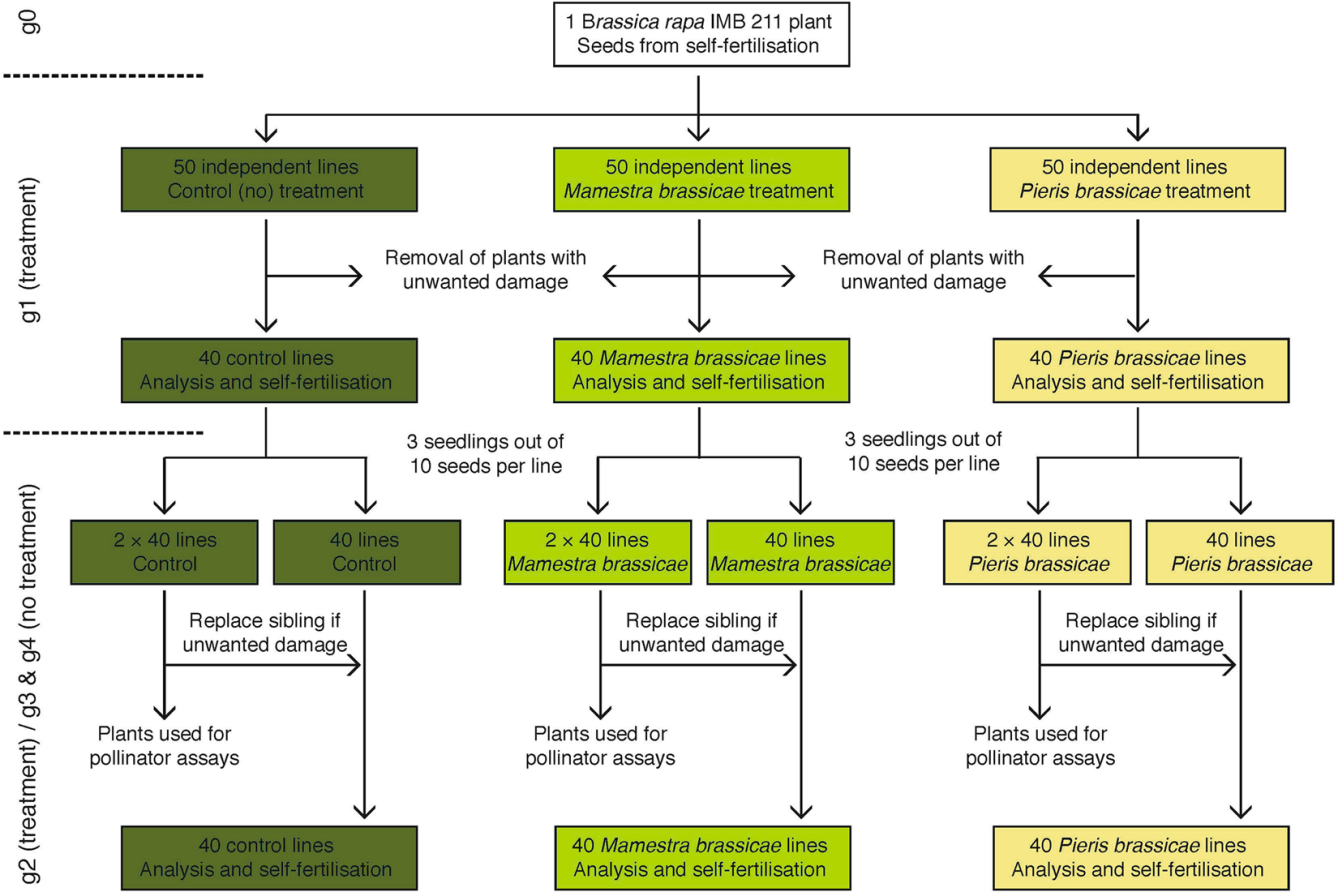
Propagation scheme showing the initial plant (g0), the two generations with herbivore treatment (g1 and g2) and the two generations without herbivory (g3 and g4) of this experiment.

To minimize the presence of standing genetic and epigenetic variation that could mask *de novo* phenotypic changes, we used seeds from a single individual of the self-fertilizing inbred *Brassica rapa* line IMB 211^54^ for the first generation (Fig. 4, see Whittle *et al*.^10^ for a similar setup). Plants were grown in 7 × 7 cm pots with standardized soil (Einheitserde Werkverband e.V., Germany) and randomized daily in a climate chamber (24 h light, 21 °C, 65% relative humidity, daily watering). Fourteen days after germination, a total of 150 plants were randomly assigned either to a control, *Mamestra brassicae* or *Pieris brassicae* group (Fig. 4). Two first instar *M. brassicae* or *P. brassicae* larvae obtained from an in-house breeding were placed each on the newest (fourth) mature leaf of plants from the corresponding group and allowed to feed for seven days (ca. 1–2 days before anthesis; control plants were left untreated). Aiming at damage of ca. 25% of total leaf tissue per plant, inactive larvae and larvae reaching the third instar were replaced with new ones as needed. Plants with damaged reproductive parts (flower stalks and buds) were removed and the final sample size was balanced to 120 plants (40 plants per group, Fig. 4). Seven days after herbivore removal, all plants were transferred to a greenhouse table under an insect net and kept under the same conditions as above until seed ripening.

For the second generation, ten seeds from each of the 120 plants were sown, germination rate was recorded from these seeds and three seedlings per parent were chosen, resulting in a total of 360 plants (3 × 40 plants per group, Fig. 4). Plants received the same conditions and treatments as in generation 1. After herbivore removal, plants with damaged reproductive parts (flowers and stalks) were excluded and one of the remaining descendants per parent was randomly selected to again balance the sample size to 120 plants (40 plants per group). The other plants were used for pollinator bioassays (see below). After generation 2, herbivore treatment was ceased and the plants were grown for two more generations (generations 3 and 4) under the same environmental conditions and propagation regime (Fig. 4).

### Morphological analysis

Phenotypic analysis was conducted at four different time points: 1. leaf VOCs were collected from 20 randomly selected plants per group 48 h after the onset of herbivory. 2. flower VOCs as well as all phenotypic measurements (except nectar and seed traits) were collected from all plants individually three days after anthesis. Plant height, mean petal surface of three flowers (4 petals × π × 0.5 petal width × 0.5 petal length) and number of leaves, buds and open flowers were recorded. 3. mean nectar production of three flowers was measured for all plants with 5 μl glass capillaries six days after anthesis. 4. number of siliques and mean seed weight of ten seeds were recorded from all plants after seed ripening. Total seed weight was further used to estimate the total amount of seeds per plant.

### Volatile analysis

Volatiles were collected from leaves and inflorescences with non-destructive headspace sorption. Whole plants (leaf VOC) or inflorescences (flower VOC) were enclosed in Sigmacote-treated glass cylinders (Sigma Aldrich, Switzerland) and sealed with aluminium foil (leaf VOC) or Teflon plates around the peduncle (flower VOC). VOCs were sampled by simultaneously pushing clean air through active charcoal filters into the cylinders and sucking air out through glass tubes loaded with 20 mg Tenax TA (60/80 mesh) at a flow rate of 100 ml min^−1^ for 2 h. Background VOC levels were determined with samples from empty glass cylinders. Analysis of VOC samples was performed using gas chromatography with mass selective detection (GC-MSD) as described in Schiestl *et al*.^26^. Identification and quantification of compounds was achieved with a mass spectral library built on calibration curves using three to five different concentrations of authentic reference standards^26^. Non-identifiable VOC as well as VOC with an amount below the mean background level in >10% of samples were excluded from the dataset. VOC quantities were converted to pg flower^−1^ l^−1^ sampled air. All analyses were done using the Agilent MSD ChemStation program E. 02.02 (2011).

### Bioassays with parasitoids

Between 24 and 48 h after the onset of herbivory treatment, five (in generations 1 and 4) or six plants (in generation 3) each from the control and treatment groups were randomly selected and brought to a laboratory at the University of Neuchâtel, Switzerland. Plants from generation 2 were mechanically damaged during transport and thus could not be used for this assay. The herbivores were removed and the soil was covered with aluminium foil around the hypocotyl. One plant per treatment group (=one set of three plants) was randomly placed in a clean four-arm olfactometer technically identical to the one described in Turlings *et al*.^55^, with a pot containing aluminium foil covered soil in the empty fourth arm. Clean air was pushed through each arm with a flow rate of 0.7 l min^−1^, converging in a central chamber, in which five mated female *Cotesia glomerata* parasitoid wasps were released. All wasps were obtained from an in-house breeding. After 30 min, the wasps were removed and their choice was recorded; wasps residing in the central chamber were counted as undecided. This procedure was repeated five times to a total of 5 × 5 wasps per plant set. Afterwards, the olfactometer was cleaned with acetone and pentane and a new plant set was installed.

### Bioassays with pollinators

The attraction of pollinators to herbivory-treated plants was determined in dual-choice bioassays with bumblebees (*Bombus terrestris*, Andermatt Biocontrol, Andermatt, Switzerland). The assays were conducted with excess plants from generations 2, 3 and 4 (Fig. 4, see above) five days after onset of anthesis, corresponding to six to seven days after herbivore removal. Before the assay, the bees were allowed to forage on untreated *B. rapa* plants for 2 h. Subsequently, one pair consisting of a randomly chosen control and a either an *M. brassicae* or a *P. brassicae*-treated plant was placed with 20 cm distance in a flight cage (2.5 m length, 1.8 m width, 1.2 m height). Bees were released individually in the cage. After the first landing on a flower, the chosen plant was recorded and the bee was removed from the experiment. After a sequence of five visits, all bees were returned to their hive box, the plant pair was removed and a new pair was installed switching the position of control- and treated plant. A total of 15 plant pairs per herbivore group and generation were used in this experiment.

### Plant trait statistics

Prior to the statistical analysis, all response variables were Box-Cox transformed^56^ and tested for normality and homoscedasticity with a Shapiro-Wilk test^57^ and Fligner-Killeen’s test^58^ respectively. Response variables were assigned to a morphological traits class, reproductive traits class, leaf VOC class and floral VOC class. Effects on trait classes were analysed for each plant generation individually with a two-way MANOVA with treatment and sampling day (resulting from time to anthesis) as explanatory variables. Treatment effects on traits within significant MANOVA classes were calculated using a two-way ANOVA with holm adjustment of all *P*-values within this plant generation and comparisons between treatments were performed with *post hoc* Tukey HSD tests. Finally, all response variables (except total petal area and leaf VOCs) were ranked within generation, z-transformed across all generations and overall treatment effects were assessed with linear discriminant analysis (LDA). For classification, both generation and treatment were combined in one grouping factor (4 × 3 = 12 classes) and the discriminatory power was tested using Wilks’ Λ. Statistical analyses were carried out in *R* v. 3.0.2 (R Development Core Team 2013) with the packages *MASS* v. 7.3–47 (Venables and Ripley 2002) and *caret* v. 6.0–76 (Kuhn 2008).

### Bioassay statistics

As suggested by Turlings *et al*.^55^, we analysed preferences of *C. glomerata* wasps with a negative binomial mixed model adjusting for zero inflation and overdispersion. Treatment was included as fixed effect and the interaction between treatment and plant group as random effect. In generation 1, none of the wasps chose the empty arm, which lead to complete separation of the random term in the model. We thus restricted our analysis to the three arms containing control and treated plants to enable a comparison between plant generations. However, we additionally fitted models including all four arms for generations 3 and 4 to assess choice difference between all arms within those generations. For all models, comparisons between treatments were performed with *post hoc* Tukey HSD tests. The effects of *P. brassicae* and *M. brassicae* herbivory on pollinator attraction were calculated using a binomial test. Statistical analyses were carried out in *R* with the package *glmmADMB* v 0.8.3.3 (Bolker et al. 2012).

### Data availability

The datasets generated during and/or analysed during the current study are available at 10.5167/uzh-149519.

## Electronic supplementary material

Supplementary Material
