# Supplementary material for: Trans-generational inheritance of herbivory-induced phenotypic changes in Brassica rapa
Source: Sci Rep. 2018 Feb 23;8:3536. doi: 10.1038/s41598-018-21880-2 (PMC5824794; doi:10.1038/s41598-018-21880-2)
Supplement: Supplementary file 1 — Supplementary Material [file 41598_2018_21880_MOESM1_ESM.doc]

Supplementary Material

**Trans-generational inheritance of herbivory-induced phenotypic changes in *Brassica rapa***

**Roman T. Kellenberger^1*^, Gaylord A. Desurmont^2,3^, Philipp M. Schlüter^1^, Florian P. Schiestl^1^**

1: Department of Systematic and Evolutionary Botany, University of Zurich, Zollikerstrasse 107, CH-8008 Zurich, Switzerland

2: Institute of Biology, University of Neuchâtel, Avenue du 1er-Mars 26, CH-2000 Neuchâtel, Switzerland

3: EBCL USDA ARS, Campus international de Baillarguet, 34980 Montferrier sur lez, France

*** Correspondence:**

Roman T. Kellenberger

roman.kellenberger@uzh.ch

# Supplementary Figures

**Figure S1.** Boxplots showing treatment effects on all measured phenotypic traits across all four plant generations (Red diamonds denoting group means). **(a) to (g)** show morphological traits, **(h) to (k)** show reproductive traits, **(l) to (n)** show leaf VOC, and **(o) to (v)** show flower VOC. Letters above bars indicate significant trait differences between treatment groups in this plant generation (two-way ANOVA with *post hoc* Tukey HSD).

# Supplementary Tables

**Table S1.** Both mean and ± 1 standard error (SE) are shown for each treatment group per trait (all morphological traits as well as all leaf and floral VOC) and generation (both g1 and g2 with direct treatment as well as g3 with short retaining effects, and g4 with long retaining effects)

|  | Treatment g1 Mean ± 1SE | | | Treatment g2 Mean ± 1SE | | | Short retaining g3 Mean ± 1SE | | | Long retaining g4 Mean ± 1SE | | |
| --- | --- | --- | --- | --- | --- | --- | --- | --- | --- | --- | --- | --- |
|  | Control | *Mamestra* | *Pieris* | Control | *Mamestra* | *Pieris* | Control | *Mamestra* | *Pieris* | Control | *Mamestra* | *Pieris* |
| **Morphological traits** |  |  |  |  |  |  |  |  |  |  |  |  |
| Plant height [cm] | 17.2 ± 0.3 | 16.3 ± 0.2 | 15.9 ± 0.3 | 15.2 ± 0.3 | 15.3 ± 0.3 | 14.4 ± 0.3 | 13.7 ± 0.3 | 13.9 ± 0.3 | 13.1 ± 0.3 | 17.3 ± 0.3 | 17.1 ± 0.3 | 17.1 ± 0.3 |
| Flower number | 7.4 ± 0.2 | 6.7 ± 0.3 | 6.8 ± 0.2 | 5.9 ± 0.2 | 6.1 ± 0.2 | 5.5 ± 0.2 | 5.6 ± 0.2 | 5.6 ± 0.2 | 5.0 ± 0.2 | 8.2 ± 0.2 | 8.6 ± 0.3 | 8.5 ± 0.2 |
| Bud number | 101.1 ± 2.9 | 101.9 ± 3.0 | 97.6 ± 3.3 | 75.2 ± 2.2 | 85.0 ± 2.5 | 71.1 ± 2.8 | 71.1 ± 2.5 | 87.2 ± 2.3 | 72.2 ± 2.8 | 83.4 ± 2.5 | 72.5 ± 2.8 | 71.3 ± 2.5 |
| Leaf number | 19.5 ± 0.5 | 19.0 ± 0.5 | 19.0 ± 0.5 | 16.1 ± 0.3 | 16.5 ± 0.2 | 16.5 ± 0.3 | 16.5 ± 0.4 | 19.7 ± 0.4 | 17.2 ± 0.5 | 19.0 ± 0.4 | 17.3 ± 0.4 | 17.3 ± 0.5 |
| Nectar [nl/3 flowers] | 221.7 ± 12.2 | 175.4 ± 11.4 | 150.9 ± 10.1 | 233.9 ± 11.7 | 166.3 ± 13.2 | 156.2 ± 10.6 | 144.4 ± 10.8 | 207.5 ± 14.4 | 145.5 ± 10.2 | 211.3 ± 11.3 | 141.8 ± 11.2 | 141.6 ± 9.0 |
| Petal area/flower [mm^2^] | 57.8 ± 0.7 | 45.4 ± 0.9 | 45.0 ± 0.9 | 40.4 ± 1.1 | 31.7 ± 0.9 | 28.8 ± 0.8 | 44.2 ± 0.9 | 34.8 ± 0.7 | 39.1 ± 0.9 | 38.7 ± 0.7 | 39.3 ± 0.7 | 38.0 ± 0.8 |
| Total petal area [mm^2^] | 428.2 ± 12.7 | 305.2 ± 16.0 | 307.0 ± 11.7 | 242.5 ± 11.8 | 198.1 ± 9.9 | 163.3 ± 10.5 | 245.7 ± 9.7 | 196.2 ± 8.3 | 195.7 ± 9.9 | 317.9 ± 10.2 | 338.6 ± 12.2 | 322.1 ± 11.8 |
| **Reproductive traits** |  |  |  |  |  |  |  |  |  |  |  |  |
| Silique number | 82.9 ± 2.3 | 78.6 ± 1.5 | 77.2 ± 2.0 | 84.5 ± 2.3 | 80.7 ± 2.4 | 79.2 ± 2.7 | 74.3 ± 2.8 | 88.9 ± 2.3 | 84.2 ± 2.6 | 48.0 ± 1.3 | 56.1 ± 2.2 | 61.0 ± 2.3 |
| Seed weight [mg/10 seeds] | 13.1 ± 0.2 | 12.8 ± 0.2 | 13.1 ± 0.2 | 11.7 ± 0.3 | 11.4 ± 0.3 | 10.4 ± 0.3 | 14.9 ± 0.2 | 15.8 ± 0.2 | 15.5 ± 0.2 | 16.5 ± 0.1 | 15.3 ± 0.4 | 15.5 ± 0.3 |
| Seed number | 641.6 ± 16.5 | 626.3 ± 14.4 | 601.7 ± 13.0 | 523.6 ± 23.0 | 488.4 ± 19.9 | 448.5 ± 22.5 | 328.3 ± 15.1 | 409.4 ± 12.6 | 370.1 ± 20.3 | 263.6 ± 9.9 | 307.2 ± 15.1 | 314.3 ± 13.8 |
| Seed viability [%] | 100.0 ± 0.0 | 100.0 ± 0.0 | 99.2 ± 0.6 | 94.5 ± 2.3 | 83.5 ± 3.6 | 94.7 ± 2.5 | 51.8 ± 3.3 | 61.8 ± 2.9 | 52.9 ± 4.0 | 96.8 ± 1.0 | 97.4 ± 2.1 | 98.4 ± 0.7 |
| **Leaf VOC [pg/ l]** |  |  |  |  |  |  |  |  |  |  |  |  |
| 1-Butene-4-isothiocyanate | 261.7 ± 47.5 | 580.9 ± 128.7 | 295.4 ± 57.2 | 629.1 ± 114.6 | 1898.5 ± 334.7 | 901.2 ± 91.3 | 363.2 ± 75.2 | 434.5 ± 91.7 | 351.2 ± 38.8 | 1012.9 ± 246.3 | 318.6 ± 52.8 | 568.1 ± 206.7 |
| Benzyl nitrile | 23.5 ± 1.9 | 64.9 ± 17.4 | 88.9 ± 23.9 | 131.2 ± 8.8 | 176.6 ± 9.9 | 218.4 ± 18.8 | 45.2 ± 5.2 | 52.6 ± 8.3 | 59.3 ± 9.4 | 86.7 ± 8.1 | 68.5 ± 3.8 | 79.7 ± 7.3 |
| E-α-Farnesene | 47.4 ± 4.0 | 196.9 ± 27.9 | 161.2 ± 21.8 | 210.3 ± 15.1 | 387.8 ± 24.5 | 340.8 ± 22.2 | 74.3 ± 10.4 | 94.3 ± 11.5 | 88.7 ± 7.8 | 82.0 ± 4.3 | 69.3 ± 4.3 | 91.0 ± 12.3 |
| **Flower VOC [pg/flower*l]** |  |  |  |  |  |  |  |  |  |  |  |  |
| Benzaldehyde | 336.0 ± 18.9 | 427.8 ± 27.9 | 416.8 ± 27.0 | 1522.3 ± 64.5 | 1540.8 ± 60.5 | 1785.6 ± 98.4 | 855.9 ± 44.4 | 805.0 ± 45.8 | 848.7 ± 53.1 | 1116.6 ± 35.3 | 1009.7 ± 30.1 | 1046.6 ± 28.0 |
| 1,3,5-Trimethylbenzene | 25.8 ± 1.3 | 34.7 ± 2.6 | 31.0 ± 1.8 | 127.5 ± 5.4 | 129.6 ± 5.6 | 147.3 ± 7.8 | 65.2 ± 3.6 | 62.1 ± 3.8 | 69.1 ± 4.1 | 72.4 ± 1.9 | 65.9 ± 1.8 | 69.1 ± 1.9 |
| Phenylacetaldehyde | 226.2 ± 18.1 | 170.2 ± 17.7 | 180.0 ± 14.8 | 183.7 ± 16.8 | 176.8 ± 13.1 | 161.2 ± 13.1 | 347.0 ± 47.8 | 254.6 ± 23.0 | 304.9 ± 41.5 | 266.1 ± 26.7 | 243.5 ± 16.8 | 231.4 ± 21.6 |
| Methyl benzoate | 98.4 ± 4.7 | 82.2 ± 4.4 | 82.4 ± 3.9 | 118.2 ± 6.5 | 107.1 ± 5.2 | 98.8 ± 5.3 | 84.6 ± 5.8 | 88.4 ± 5.5 | 90.4 ± 5.9 | 111.3 ± 8.3 | 98.1 ± 6.0 | 83.4 ± 5.6 |
| Benzyl nitrile | 144.9 ± 10.9 | 103.4 ± 7.1 | 108.8 ± 8.7 | 92.6 ± 8.5 | 76.3 ± 6.2 | 57.1 ± 4.9 | 62.3 ± 7.2 | 73.0 ± 7.9 | 59.2 ± 5.6 | 128.7 ± 12.0 | 143.3 ± 9.4 | 110.7 ± 7.9 |
| Methyl salicylate | 8.8 ± 0.4 | 6.6 ± 0.4 | 6.3 ± 0.3 | 8.9 ± 0.7 | 7.3 ± 0.5 | 6.9 ± 0.5 | 9.4 ± 0.8 | 11.8 ± 1.1 | 10.6 ± 0.8 | 13.6 ± 0.9 | 9.7 ± 0.7 | 8.1 ± 0.5 |
| Z-α-Farnesene | 45.2 ± 2.4 | 38.8 ± 1.9 | 37.8 ± 2.0 | 125.0 ± 6.3 | 122.0 ± 6.6 | 126.6 ± 7.4 | 102.0 ± 7.7 | 97.8 ± 6.3 | 97.7 ± 7.1 | 58.3 ± 3.4 | 50.2 ± 2.4 | 49.3 ± 2.3 |
| E-α-Farnesene | 791.0 ± 40.9 | 586.9 ± 33.2 | 622.2 ± 33.0 | 813.5 ± 62.0 | 717.9 ± 47.7 | 580.2 ± 38.1 | 978.8 ± 74.4 | 853.9 ± 55.3 | 962.3 ± 68.5 | 1042.0 ± 68.9 | 879.8 ± 57.5 | 832.7 ±54.1 |

**Table S2.** Loading values of the first and second linear discriminant function (DF1 and DF2) separating plant generations and treatment groups. DF1 explains 44.0%, and DF2 explains 23.3% of the variance between groups. Total petal area was excluded from the analysis due to collinearity with petal area. Also, leaf VOCs could not be included as they were not available for all plants.

|  | Loading values of DF1 | Loading values of DF2 |
| --- | --- | --- |
| **Morphological traits** |  |  |
| Plant height [cm] | -0.076079773 | -0.175217817 |
| Flower number | 0.008781509 | -0.092859298 |
| Bud number | 0.423497181 | 0.273369065 |
| Leaf number | 0.176404615 | 0.229943638 |
| Nectar [nl/3 flowers] | -0.145747262 | 0.637136433 |
| Petal area/flower [mm^2^] | -1.28181005 | -0.09198888 |
| Total petal area [mm^2^] | n.a. | n.a. |
| **Reproductive traits** |  |  |
| Silique number | -0.04760028 | -0.295756535 |
| Seed weight [mg/10 seeds] | 0.060193161 | 0.33116108 |
| Seed number | 0.154670424 | 0.046064437 |
| Seed viability [%] | 0.04704356 | 0.033367515 |
| **Leaf VOC [pg/ l]** |  |  |
| 1-Butene-4-isothiocyanate | n.a. | n.a. |
| Benzyl nitrile | n.a. | n.a. |
| E-α-Farnesene | n.a. | n.a. |
| **Flower VOC [pg/flower*l]** |  |  |
| Benzaldehyde | -0.059678475 | -0.40739848 |
| 1,3,5-Trimethylbenzene | 0.140416673 | 0.206667465 |
| Phenylacetaldehyde | 0.08577066 | -0.253077722 |
| Methyl benzoate | -0.040703581 | -0.028593748 |
| Benzyl nitrile | 0.083348219 | 0.001104192 |
| Methyl salicylate | -0.087293613 | 0.659169218 |
| Z-α-Farnesene | -0.036769696 | -0.11862906 |
| E-α-Farnesene | 0.08250177 | 0.215556585 |
